# Integration of genomic analysis and transcript expression of ABCC8 and KCNJ11 in focal form of congenital hyperinsulinism

**DOI:** 10.3389/fendo.2022.1015244

**Published:** 2022-10-21

**Authors:** Ilse Wieland, Ina Schanze, Ina Marianti Felgendreher, Winfried Barthlen, Silke Vogelgesang, Klaus Mohnike, Martin Zenker

**Affiliations:** ^1^ Institute of Human Genetics, University Hospital Otto-von-Guericke- University Magdeburg, Magdeburg, Germany; ^2^ Department of Pediatric Surgery, Protestant Hospital of Bethel Foundation, University Hospital OWL, University of Bielefeld, Bielefeld, Germany; ^3^ University Medicine, Institute of Pathology, University of Greifswald, Greifswald, Germany; ^4^ Dept of Pediatrics, University Hospital Otto-von-Guericke-University Magdeburg, Magdeburg, Germany

**Keywords:** *ABCC8*, Achaete-scute complex homolog 2, CHI, *KCNJ11*, UPD 11p

## Abstract

**Background:**

The focal form of CHI is caused by an autosomal recessive pathogenic variant affecting the paternal homologue of genes *ABCC8* or *KCNJ11* and a second somatic event specifically occurring in the affected islet of Langerhans. The approach of this study was to integrate the genetic changes occurring in pancreatic focal lesions of CHI at the genomic and transcriptional level.

**Research Design and Methods:**

Patients receiving therapeutic surgery and with proven *ABCC8* or *KCNJ11* pathogenic variants were selected and analyzed for loss of heterozygosity (LOH), changes in copy number and uniparental disomy (UPD) on the short am of chromosome 11 by molecular microarray analysis and methylation-specific MLPA. Gene expression was analyzed by RT-PCR and Massive Analysis of cDNA Ends (MACE).

**Results:**

Both genes, *ABCC8* and *KCNJ11*, are located in proximity to the Beckwith-Wiedemann (BWS) imprinting control region on chromosome 11p15. Somatic paternal uniparental isodisomy (UPD) at chromosome 11p was identified as second genetic event in focal lesions resulting in LOH and monoallelic expression of the mutated *ABCC8/KCNJ11* alleles. Of five patients with samples available for microarray analysis, the breakpoints of UPD on chromosome 11p were different. Samples of two patients were analyzed further for changes in gene expression. Profound downregulation of growth suppressing genes *CDKN1* and *H19* was detected in focal lesions whereas growth promoting gene *ASCL2* and pancreatic transcription factors of the endocrine cell lineage were upregulated.

**Conclusions:**

Paternal UPD on the short arm of chromosome 11 appears to be the major second genetic event specifically within focal lesions of CHI but no common breakpoint for UDP can be delineated. We show for the first time upregulation of growth promoting *ASCL2* (achaete-scute homolog 2) suggestive of a driving factor in postnatal focal expansion in addition to downregulation of growth suppressing genes *CDKN1C* and *H19*.

## Introduction

Congenital hyperinsulinism (CHI) causes persistent hypoglycemia due to uncontrolled insulin secretion in newborns and infants (1, 2). The most severe form of CHI is caused by inactivating pathogenic variants in the *ABCC8* (MIM 600509) and *KCNJ11* (MIM 600937) genes encoding subunits SUR1 and Kir6.2 of the ATP-sensitive K(+) channel (3, 4). This channel is primarily expressed in the pancreas and to a much lesser degree in other tissues. Genetic testing in several larger patient cohorts revealed more than 100 pathogenic variants in these genes. Most of them are small pathogenic variants detectable by standard sequence analysis (5). To improve patient care, genetic testing has now been implemented in clinical management of CHI (6, 7).

Two major clinical forms of CHI are known. The diffuse form affects all ß-cells in the pancreas and is mainly caused by biallelic recessive inheritance of inactivating pathogenic variants and less frequently by dominantly acting pathogenic variants. Medical therapy, frequent feeding and subtotal pancreatectomy are the current treatment regimens for the diffuse form (8). The focal form is characterized by ß-cell hyperplasia in an affected islet of Langerhans within the pancreas. Surgical resection of a focal lesion potentially cures the patient (9, 10). The focal form appears to be caused by an autosomal recessive pathogenic variant affecting the paternal homologue of either the *ABCC8* or *KCNJ11* gene combined with somatic loss of heterozygosity (LOH) in the lesion (11–13).

*ABCC8* and *KCNJ11* are neighboring genes and they are located on the short arm of chromosome 11 in region 11p15 proximal to the imprinting region that when disrupted causes imprinting disorders Beckwith-Wiedemann syndrome (BWS) and Russell-Silver syndrome (RSS). The critical genomic imprinting region is responsible for the expression of growth regulatory genes depending on the parental origin (14, 15). The *IGF2* gene (MIM 147470) encoding insulin-like growth factor 2 is expressed from the paternally derived chromosome and functions as growth promoting factor during embryogenesis and fetal development. The non-coding RNA of the *H19* gene (MIM 103280) is expressed from the maternally derived chromosome and it is a negative regulator of *IGF2* and other genes (16–18). Likewise, the *CDKN1C* gene (MIM 600856) encoding the inhibitor of G1 cyclin dependent kinases p57^kip2^, is preferentially expressed from the maternal allele. In BWS the clinical features are variable manifestations of macrosomia, visceromegaly of intra-abdominal organs and additional features including hyperinsulinism in a small amount of patients. However, BWS patients usually do not carry pathogenic variants in either *ABCC8* or *KCNJ11* and the underlying mechanism responsible for hyperinsulinism in these patients is not known. In BWS, expression from the maternal chromosome 11p15.5 is compromised either by imprinting defects or by paternal uniparental isodisomy (UPD) in 25% of patients and copy number variations (CNVs) in 9% of patients (14, 19). The focal form of CHI appears to represent a highly restricted type of UPD11p15 somatic mosaicism. In focal lesions LOH at 11p15 was first described using microsatellites and was later confirmed by loss of the maternal allele (12, 20, 21). Further studies showed an imbalance of gene expression in focal lesions (22).

In this study, we performed an integrative analysis of LOH, copy number changes and methylation at the BWS/RSS region followed by changes in gene expression in focal pancreatic lesions of patients harboring pathogenic variants in *ABCC8* and *KCNJ11*, respectively.

## Materials and methods

### Patients and pancreatic tissue samples

Patients were from the German Registry for Congenital Hyperinsulinism (23). Written informed consent was obtained from the parents of patients and in accordance to the approval by the local ethics committees. Patients were treated by surgical therapy because of the clinical and genetic indication of focal CHI and localization of lesions by imaging diagnostics (10). Histological examination of resected pancreatic tissue presented with a lobular structure. Focal accumulation of atypical islet cells of Langerhans showed only a small rim of adjacent exocrine parenchyma. The endocrine cells exhibited huge nuclei and a broad eosinophilic cytoplasm. By immunohistochemistry the endocrine cells showed a strong positive reaction with insulin, whereas no reaction with p57 antibody was observed.

### Mutation/variant analysis

Following histological examination of resected pancreatic lesions DNA and RNA was simultaneously extracted from deep frozen tissues using the QIAamp DNeasy Blood & Tissue Kit and QIAmp RNeasy Mini Kit (QIAGEN GmbH, Hilden, Germany).

Pathogenic variants and LOH in genes *ABCC8* and *KCNJ11* were analyzed by PCR amplification of the corresponding exons followed by Sanger sequencing using the Big Dye Terminator Cycle Sequencing kit and ABI 3500XL sequencer (Applied Biosystems, Foster City, USA). Coding SNPs in *ABCC8* (dbSNP, NCBI) rs1799858 (exon 14), rs1805036 (exon 21), rs1799859 (exon 31), rs757110 (exon 33) and in *KCNJ11* rs5213, rs5215, rs5218, rs5219 were also included in LOH analysis.

Sequences were processed by SeqPilot software 4.2.1 (JSI Medical Systems GmbH, Ettenheim, Germany). The sequencing data were compared with reference sequence NM_001287174.1 (ENST00000302539; *ABCC8*) and NM_000525.3 (ENST00000339994; *KCNJ11*).

### Methylation-specific MLPA

Methylation-specific (MS) Multiplex Ligation-dependent Probe amplification (MLPA) analysis was carried out using probemix ME030-C3 as described by the manufacturer (MRC Holland, Amsterdam, The Netherlands). Amplification products were identified and quantified by capillary electrophoresis on an ABI 3500XL genetic analyzer. MLPA profiles were analyzed with the module MLPA of SeqPilot (JSI Medical Systems GmbH, Ettenheim, Germany).

### Molecular microarray analysis

SNP-based chromosomal microarray (CMA) analysis was performed using a CytoScan™ HD microarray and the Chromosome Analysis Suite v4.3.0.71 (Thermo Fisher Scientific, Waltham, MA USA). All genomic positions were according to the GRCh37/hg19 build of the human reference genome.

### RT-PCR, massive analysis of cDNA ends (MACE)

Expression analysis of *ABCC8* and *KCNJ11* was performed by RT-PCR. The same pancreatic tissue samples were examined for genomic and RNA changes. Total RNA was reverse transcribed using the Super ScriptIII™ RT-PCR kit (Invitrogen, Carlsbad, USA) as recommended by the supplier. RT-PCR was performed with primers from the coding regions of the genes using internal exon-spanning primers for *ABCC8*. For the single exon gene *KCNJ11* the RNA was treated with DNAse prior to reverse transcription in order to exclude amplification of residual genomic DNA in the reaction. For controlling specific transcript amplification of *KCNJ11*, coding SNPs of *ABCC8* were analyzed in parallel by exon-spanning RT-PCR. RT-PCR products were analyzed by direct sequencing as outlined above.

MACE was performed on extracted RNA from focal lesions and adjacent pancreatic normal tissue of the same patients (Patient 3 and 8) and expression was compared within the same patient. Data were analyzed as described previously (24) by GenXPro GmbH, Frankfurt a.M., Germany.

## Results

### Pathogenic variants in K_ATP_-channel genes *ABCC8* and *KCNJ11*

Paternally transmitted heterozygous pathogenic variants in either *ABCC8* or *KCNJ11* were previously identified during molecular genetic analysis in blood cells of the patients and their parents and have been described by Mohnike et al. (23) and Barthlen et al. (10). Of the 10 patients included in this study, 6 harbored pathogenic variants in *ABCC8* and 4 harbored pathogenic variants in *KCNJ11* (**Table 1**).

**Table 1:** Genetic and expression analysis of pancreatic lesions from focal CHI.

**Patient**	**Exon**	**Pathogenic Variant** **Nucleotide Protein**		**Observed freq. [Ref.]**	**Age at surgery (months)**	**mRNA expression**	**LOH**	**Paternal UPD11p15**
***ABCC8***								
**1**	1	c.50T>C	p.Val17Ala	2†	10	monoallelic mutant	++	++
**2**	10	c.1530G>T	p.Lys510Asn	1	10	monoallelic mutant	++	++
**3**	12	c.1792C>T	p.(Arg598*)	Multiple [CM050968]	7	no (NMD)	++	++
**4**	34	c.4162_4164delTTC	p.Phe1388del	Multiple [CD962164]	9	monoallelic mutant	++	++
**5**	35	c.4241C>T	p.Pro1414Leu	Multiple [CM068331]	6	monoallelic mutant	++	++
**6**	35	c.4259C>T	p.Ser1420Leu	1	2	monallelic mutant	+	+
***KCNJ11***								
**7**	1	c.286G>A	p.Ala96Thr	1†	2	mutant/wt 75%/25%	+	+
**8**	1	c.612C>A	p.Asp204Glu	2 [CM083531]	2	monoallelic mutant	++	++
**9**	1	c.844G>A	p.Glu282Lys	3 [CM071810]	17	monoallelic mutant	++	++
**10**	1	c.901C>G	p.Arg301Gly	Multiple [CM088147]	6	monoallelic mutant	(+)	+

Patients described in Mohnike et al. (23) and Barthlen et al. (10).

Pathogenic variants were of paternal origin and heterozygous in blood or adjacent pancreatic tissue.

ABCC8 RefSeq NM_001287174.1, KCNJ11 RefSeq NM_000525.3.

^†^ABCC8 c.50T>C was recorded once in ExAC (11:17498274 A/G), allele frequency 1.297e-05; KCNJ11 c.286G>A was recorded once in ExAC (11:17409353 C/T), allele frequency of 8.269e-06

[Ref.] pathogenic variants reported in Human Gene Mutation Database (HGMD).

LOH, loss of heterozygosity; ++, >80-100% loss of the maternal allele; +, >50-80% loss of the maternal allele; (+), >20-50% loss of the maternal allele; UPD 11p15, uniparental isodisomy including paternal imprint; ++, complete pUPD; +, incomplete pUPD.

The DNA of pancreatic samples was analyzed for LOH following histological examination of frozen tissue biopsies. Focal lesions and adjacent normal pancreatic tissue if available were examined. LOH analysis performed at the pathogenic variant site in the respective sample in addition to informative intragenic single nucleotide polymorphisms (SNPs) of both, *ABCC8* and *KCNJ11*, genes was close to 100% in 7 samples (**Table 1**).

### Paternal uniparental isodisomy in focal lesions

**Figure 1:**
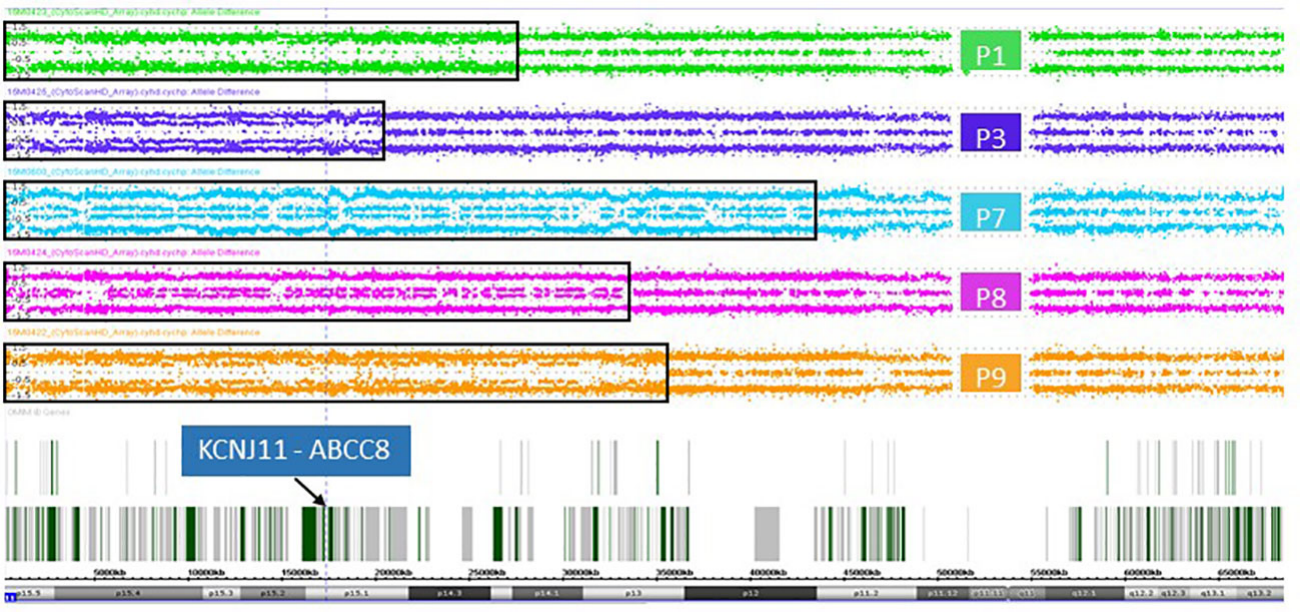
Allele difference plots of chromosome 11 on DNA extracted from pancreatic lesions of patient 1 (green panel), patient 3 (purple panel), patient 7 (blue panel), patient 8 (magenta panel) and patient 9 (orange panel) show a mosaic pattern of segmental uniparental disomy (UPD) of the short arm of chromosome 11 of different sizes. The extent of the UPD regions indicated by the black rectangles. The vertical dotted line and the arrow indicates the location of the genes *KCNJ11* and *ABCC8*, which are located within the UPD region in all five patient samples.

Copy number-neutral LOH at the *ABCC8/KCNJ11* locus as determined by MLPA suggested uniparental isodisomy (UPD) of chromosomal region 11p15 including the *ABCC8* and *KCNJ11* loci. At both imprinting centers of the BWS/RSS region on 11p15.5, an imbalance in methylation typically found in BWS patients with paternal UPD 11p15 was detected in 7 focal samples (**Table 1**; **Figure 1**). In 3 patients (Patient 6, 7, 10) with intermediate LOH in genomic DNA, paternal UPD 11p15 was similar in pattern but differences were less pronounced than in the other samples, thus suggesting a mosaic status for UPD 11p15 in these samples.

In DNA from pancreatic tissue specimens of patients 1, 3, 7, 8, and 9 we could demonstrate regions of copy neutral loss of heterozygosity (LOH) on the short arm of chromosome 11, suggesting segmental uniparental disomy (UPD) 11. All samples showed different dimensions of the UPD region with the smallest region in patient 3 (20,3Mb) and the largest region in patient 7 (43,2Mb) (**Figure 1** and **Table 2**). In all patients the *ABCC8* gene is located within the UPD region. Furthermore the samples showed different levels of mosaicism ranging from <50% to >75%.

**Table 2:** Molecular microarray analysis.

**Patient**	**Karyotype according ISCN2020**	**Level of mosaicism**
**1**	arr[GRCh37] p15.5p14.1(1_27500235)x2 hmz	> 75%

**3**	arr[GRCh37] p15.5p15.1(1_20328840)x2 hmz	50-75%

**7**	arr[GRCh37] p15.5p12(1_43161203)x2 hmz	> 50%

**8**	arr[GRCh37] p15.5p13(1_33012493)x2 hmz	> 50%

**9**	arr[GRCh37] p15.5p13(1_35381495)x2 hmz	50-75%

Positions were according to the GRCh37/hg19.

### Monoallelic expression of *ABCC8* and *KCNJ11* transcripts from paternally transmitted mutant alleles

**Figure 2:**
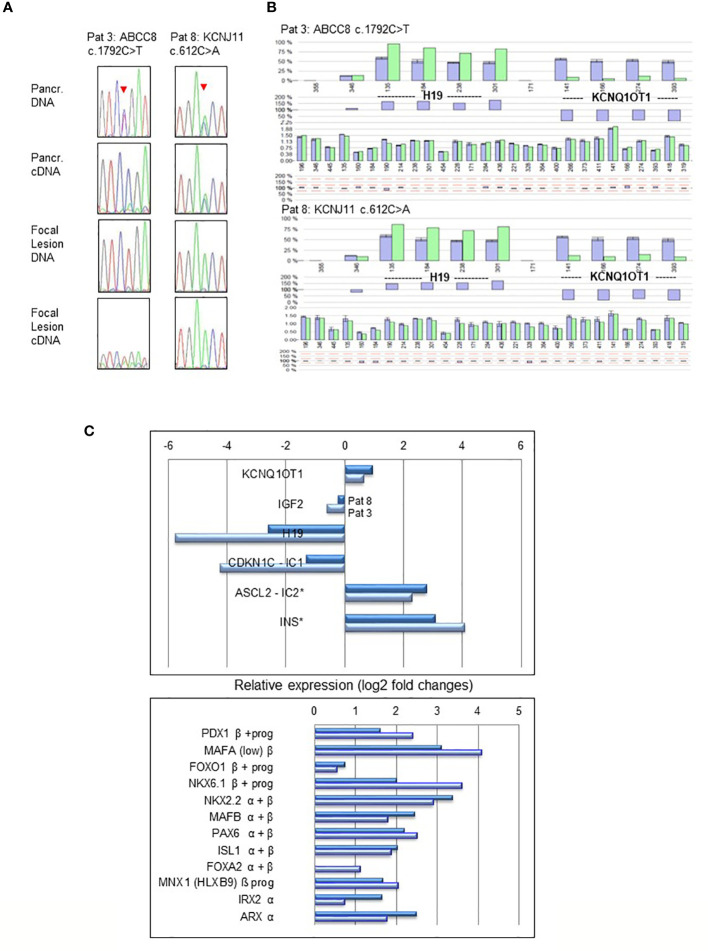
LOH and expression analysis performed in pancreatic normal tissue and focal lesions of Patient 3 (*ABCC8* c.1792C>T) and Patient 8 (*KCNJ11* c.612C>A). Heterozygosity of the pathogenic variants and biallelic expression **(A)** is evident in normal pancreatic tissue (upper panel), whereas focal lesions show monoallelic expression and LOH (lower panel). Sequencing profiles show the respective pathogenic variants in *ABCC8* and *KCNJ11* (indicated by an arrowhead) obtained from genomic DNA and reverse transcribed cDNA from focal lesion and pancreatic (Pancr.) normal tissue. In cDNA of Patients 3 pathogenic variant c.1792C>T causes NMD of the transcript resulting in lack of the respective ABCC8 transcripts. Epigenetic and copy number analysis **(B)** at the BWS/RSS imprinting region on 11p15.5 by MS-MLPA demonstrating paternal UPD11p15.5 in pancreatic focal lesions of Patient 3 (*ABCC8* c.1792C>T) and Patient 8 (*KCNJ11* c.612C>A). The pattern of hypermethylation of imprinting center 1 (IC1) at probes for *H19* and hypomethylation of IC2 at probes for *KCNQ1OT1* indicate paternal UPD11p15.5 typically found in BWS with UPD. Dark blue bars are results of three controls compared to the focal DNA (light green bars). Differences of controls and patient DNA shown below each profile. Relative gene expression (log2-fold changes) of BWS/RSS-Region 11p15 (upper panel) and pancreatic transcription factors (lower panel) by MACE analysis in focal lesions of Patient 3 (light blue) and Patient 8 (dark blue) compared to non-lesional tissue of the same patient **(C)**. Genes *ASCL2* located at imprinting center IC2 and *INS* are both not subject to genomic imprinting in humans. Pancreatic transcription factors expressed in progenitors (prog) of the endocrine lineage and in mature α- and β-cells are indicated.

In normal pancreatic tissue biallelic expression of *ABCC8* and *KCNJ11* was found by sequencing and fragment analysis. In the normal pancreatic tissue of patient 3 only the *ABCC8* transcript from the maternal wild-type allele was detectable (**Figure 2A**). This patient harbors a paternally inherited nonsense pathogenic variant in *ABCC8* resulting in a premature termination codon that is likely to cause nonsense-mediated RNA decay (NMD). By RT-PCR the amount of mutant *ABCC8* transcripts was below limit of detection despite apparent heterozygosity at the DNA level. Accordingly, there are no *ABCC8* transcripts identified beyond background level in the focal lesion when the maternal wild-type allele is lost (**Figure 2A**, Patient 3). Monoallelic expression of the mutant transcripts encoded by the paternally inherited alleles was observed for all other focal lesions from the remaining patients in this series (**Table 1**; **Figure 2A**). Patient sample 7 showed residual expression of 10-25% of the wild-type transcript from the maternal allele concomitant with incomplete LOH in genomic DNA.

### Gene expression analysis

Expression analysis for genes located in 11p15 in focal lesions of patient 3 and 8 revealed downregulation of the imprinted gene *H19* (log2: 5.8- and 2.6-fold, resulting from 690 766 and 568 794 reads in non-lesional pancreatic tissue as compared to 12 659 and 93 624 reads in focal lesions, respectively). Likewise, we found for the *CDKN1* gene 50 766 and 27 339 reads in non-lesional pancreatic tissue compared to 2 685 and 11 102 reads in focal lesions of patients 3 and 8, respectively, resulting in changes of log2: 4.2- and 1.3-fold. On the other side, we found for *ASCL2* read numbers of 7 002 and 3 417 in non-lesional pancreatic tissue versus 34 140 and 23 683 reads in focal lesions of patient 3 and 8, respectively (**Figure 2C**). This suggests a lower expression of *ASCL2* in non-lesional tissue and upregulation of *ASCL2* (achaete-scute homolog 2, MIM 601886) relative expression in focal lesions by more than log2: 2.3- and 2.8-fold, respectively. Upregulated gene expression was also observed for several pancreatic transcription factors involved in differentiation of the endocrine cell lineages of alpha and beta cells in both samples investigated (**Figure 2C**).

## Discussion

Our comprehensive molecular analysis of native frozen pancreatic lesions from 10 focal CHI patients revealed monoallelic expression of the K_ATP_-channel genes *ABCC8* and *KCNJ11* confined to focal lesions. The unaffected pancreatic tissue showed biallelic expression from both parental alleles at about similar ratios providing further evidence that both genes are not subject to parental imprinting in the pancreas. Our results also demonstrate that the recurrent nonsense pathogenic variant c.1792C>T (p.Arg598*) leads to loss of the mutated ABCC8 transcript independent of the second genetic event presumably due to nonsense-mediated mRNA decay [NMD; (25)] as shown for patient 3. Monoallelic expression in focal lesions coincided with LOH at the DNA level. This agrees well with the concept of somatic mosaicism in focal CHI that occurred in an islet by loss of the maternal allele in a progenitor cell and subsequent massive clonal expansion of the progeny cells (22). Incomplete LOH at the DNA level in 3 samples may be explained by admixture of a small cell population with features of non-lesional pancreatic cells residing within that lesion.

In all samples showing LOH, no imbalance in copy numbers was observed on 11p15 by MLPA. This suggests that neither deletion nor duplication at 11p15 is involved in focal CHI. Methylation-sensitive MLPA, however, showed an imprinting pattern of paternal UPD. These results further support the proposed paternal isodisomic UPD 11p15 as the major second genetic event causing LOH in focal CHI (21). Breakpoint-mapping in pancreatic lesions revealed no common breakpoints but encompassed the *ABCC8* and *KCNJ11* genes in the recombination interval as expected (11, 26). Somatic segmental UPD also occurs in many types of cancers and may convey a permissive growth advantage in light of Knudson’s two-hit hypothesis. A possible mechanism proposed in formation of segmental UPD in cancerous cells has been mitotic recombination events of homologous non-sister chromatids. Alternatively, an initial deletion may be compensated by re-duplication of the homologous region from the remaining chromosomal region of the other chromosome (27). In non-neoplastic tissue, less information exists on tissue-restricted UPD contributing to disease development. The focal form of CHI represents one of few examples of a cell-type restricted segmental paternal UPD 11p resembling a BWS micromosaicism. Based on the prevalence of focal compared to diffuse CHI, the risk of focal CHI in a child who carries a paternally inherited recessive pathogenic variant in *ABCC8* or *KCNJ11* was estimated to be around 1:270 (28). This may suggest that cell and tissue restricted segmental UPD are not rare somatic genetic events in pancreatic and possibly in other tissues. In fact, late onset ß-thalassemia and sickle cell anemia are other diseases with a similar mechanism of mosaic segmental paternal isodisomy at 11p15 unmasking a pathogenic variant in the *HBB* gene followed by clonal selection of hematopoetic progenitor cells due to enhanced proliferation (29, 30).

In focal pancreatic lesions the growth suppressing imprinted genes *H19* and *CDKN1C* located in 11p15 are downregulated, while no substantial change in growth promoting *IGF2* expression was detected. However, we observed upregulation of *ASCL2* (achaete-scute homolog 2) also located in 11p15 but not subject to imprinting in humans. The gene encodes a basic helix-loop-helix transcription factor, which is target of WNT signaling in intestinal stem cells and exerts oncogenic function in cell culture (31). In a mouse model, transgenic rescue of *Ascl2* expression leads to placentomegaly associated with BWS indicating a critical role of *Ascl2* in placental overgrowth (32). Our results suggest that in fact upregulated *ASCL2* is a driver in focus formation in postnatal CHI in addition to downregulated *H19* and *CDKN1*. Currently, it is not known whether downregulated expression of *H19* or additional components are responsible for upregulation of *ASCL2* in focal lesions of the pancreas. Concomitantly, several key transcription factors of the endocrine pancreatic linages including premature stages (33) were upregulated in focal lesions.

## Conclusion

In conclusion, our results support the hypothesis of paternal UPD 11p15 in focal CHI that leads to monoallelic expression of the mutated channel genes and appears to be the major second genetic event specifically in the pancreatic endocrine lineage. Clonal expansion of focal lesions appears to be driven by upregulation of growth promoting *ASCL2* (achaete-scute homolog 2) in addition to downregulation of growth suppressing genes *CDKN1* and *H19.*

## Data availability statement

The raw data supporting the conclusions of this article will be made available by the authors, upon request.

## Ethics statement

The studies involving human participants were reviewed and approved by University Hospital Magdeburg. Written informed consent to participate in this study was provided by the participants’ legal guardian/next of kin.

## Author contributions

IW, MZ planned this study. IW, IS, IF, SV, MZ performed, analyzed and interpreted the data. WB, KM recruited and collected clinical samples. IW, IS, MZ participated in writing. All authors approved the manuscript. All authors contributed to the article and approved the submitted version.

## Acknowledgments

We thank the participants of the German Registry for Congenital Hyperinsulinism for their cooperation. Part of this work is contained in the doctoral thesis of IF.

## Conflict of interest

The authors declare that the research was conducted in the absence of any commercial or financial relationships that could be construed as a potential conflict of interest.

## Publisher’s note

All claims expressed in this article are solely those of the authors and do not necessarily represent those of their affiliated organizations, or those of the publisher, the editors and the reviewers. Any product that may be evaluated in this article, or claim that may be made by its manufacturer, is not guaranteed or endorsed by the publisher.

## References

[1] Arnoux JB Verkarre V Saint-Martin C Montravers F Brassier A Valayannopoulos V . Congenital hyperinsulinism: current trends in diagnosis and therapy. Orphanet J Rare Dis (2011) 6:63. doi: 10.1186/1750-1172-6-63 21967988 PMC3199232

[2] Stanley CA De León DD . Frontiers in diabetes. In: Monogenic hyperinsulinemic hypoglycemia disorders, vol. Vol 21. (Basel, CH: Karger) (2012). doi: 10.1159/isbn.978-3-8055-9944-3

[3] Thomas PM Cote GJ Wohllk N Haddad B Mathew PM Rabl W . Mutations in the sulfonylurea receptor gene in familial persistent hyperinsulinemic hypoglycemia of infancy. Science (1995) 268:426–9. doi: 10.1126/science.7716548 7716548

[4] Nestorowicz A Inagaki N Gonoi T Schoor KP Wilson BA Glaser B . A nonsense mutation in the inward rectifier potassium channel gene, Kir6.2, is associated with familial hyperinsulinism. Diabetes (1997) 46:1743–8. doi: 10.2337/diab.46.11.1743 9356020

[5] De Franco E Saint-Martin C Brusgaard K Knight Johnson AE Aguilar-Bryan L Bowman P . Update of variants identified in the pancreatic β-cell K_ATP_ channel genes KCNJ11 and ABCC8 in individuals with congenital hyperinsulinism and diabetes. Hum Mutat (2020) 41:884–905. doi: 10.1002/humu.23995 32027066 PMC7187370

[6] Banerjee I Avatapalle B Padidela R Stevens A Cosgrove KE Clayton PE . Integrating genetic and imaging investigations into the clinical management of congenital hyperinsulinism. Clin Endocrinol (2013) 78:803–13. doi: 10.1111/cen.12153 23347463

[7] Maiorana A Barbetti F Boiani A Rufini V Pizzoferro M Francalanci P . Focal congenital hyperinsulinism managed by medical treatment: A diagnostic algorithm based on molecular genetic screening. Clin Endocrinol (2014) 81:679–88. doi: 10.1111/cen.12400 24383515

[8] Lord K Dzata E Snider KE Gallagher PR De León DD . Clinical presentation and management of children with diffuse and focal hyperinsulinism: a review of 223 cases. J Clin Endocrinol Metab (2013) 98:E1786–1789. doi: 10.1210/jc.2013-2094 PMC3816257 24057290

[9] Laje P Stanley CA Palladino AA Becker SA Adzick NS . Pancreatic head resection and roux-en-Y pancreaticojejunostomy for the treatment of the focal form of congenital hyperinsulinism. J Pediatr Surg (2012) 47:130–5. doi: 10.1016/j.jpedsurg.2011.10.032 PMC3595012 22244405

[10] Barthlen W Varol E Emptin S Wieland I Zenker M Mohnike W . Surgery in focal congenital hyperinsulinism (CHI) – the “Hyperinsulinism Germany international” experience in 30 children. Pediatr Endocrinol Rev (2016) 14:48–56. doi: 10.17458/PER.2016.BVE 28508606

[11] Giurgea I Sempoux C Bellanné-Chantelot C Ribeiro M Hubert L Boddaert N . The knudson’s two-hit model and timing of somatic mutation may account for the phenotypic diversity of focal congenital hyperinsulinism. J Clin Endocrinol Metab (2006) 91:4118–23. doi: 10.1210/jc.2006-0397 16882742

[12] Damaj L le Lorch M Verkarre V Werl C Hubert L Nihoul-Fétéké C . Chromosome 11p15 paternal isodisomy in focal forms of neonatal hyperinsulinism. J Clin Endocrinol Metab (2008) 93:4941–7. doi: 10.1210/jc.2008-0673 18796520

[13] Gillis D . Familial hyperinsulinism. In: Adam MP Ardinger HH Pagon RA Wallace SE Bean LJH Stephens K Amemiya A , editors. GeneReviews® [Internet]. (Seattle, USA: University of Washington) (2019). p. 1993–2020.

[14] Feinberg AP . Phenotypic plasticity and epigenetics of human disease. Nature (2007) 447:433–40. doi: 10.1038/nature05919 17522677

[15] Demars J Gicquel C . Epigenetic and genetic disturbance of the imprinted 11p15 region in beckwith-wiedemann and silver-russel snydromes. Clin Genet (2012) 81:350–61. doi: 10.1111/j.1399-0004.2011.01822.x 22150955

[16] Gabory A Ripoche M-A Le Digarcher A Watrin F Ziyyat A Forné T . H19 acts as a trans regulator of the imprinted gene network controlling growth in mice. Development (2009) 136:3413–21. doi: 10.1242/dev.036061 19762426

[17] Monnier P Martinet C Pontis J Stancheva I Ait-Si-Ali S Dandolo L . H19 lncRNA controls gene expression of the imprinted gene network by recruiting MBD1. Proc Natl Acad Sci USA (2013) 110:20693–8. doi: 10.1073/pnas.1310201110 PMC3870736 24297921

[18] Wang B Suen CW Ma H Wang Y Kong L Qin D . The roles of H19 in regulating inflammation and aging. Front Immunol (2020) 11:579687. doi: 10.3389/fimmu.2020.579687 33193379 PMC7653221

[19] Begemann M Spengler S Gogiel M Grasshoff U Bonin M Betz R . Clinical significance of copy number variations in the 11p15.5 imprinting control regions: new cases and review of the literature. J Med Genet (2012) 49:547–53. doi: 10.1136/jmedgenet-2012-100967 PMC3439641 22844132

[20] De Lonlay P Fournet JC Rahier J Gross-Morand MS Poggi-Travert F Foussier V . Somatic deletion of the imprinted 11p15 region in sporadic persistent hyperinsulinemic hypoglycemia of infancy is specific of focal adenomatous hyperplasia and endorses partial pancreatectomy. J Clin Invest (1997) 100:802–7. doi: 10.1172/JCI119594 PMC508251 9259578

[21] Verkarre V Fournet JC de Lonlay P Gross-Morand MS Devillers M Rahier J . Paternal mutation of the sulfonylurea receptor (SUR1) gene and maternal loss of 11p15 imprinted genes lead to persistent hyperinsulinism in focal adenomatous hyperplasia. J Clin Invest (1998) 102:1286–91. doi: 10.1172/JCI4495 PMC508975 9769320

[22] Fournet JC Mayaud C de Lonlay P Gross-Morand MS Verkarre V Castanet M . Unbalanced expression of 11p15 imprinted genes in focal forms of congenital hyperinsulinism: association with a reduction to homozygosity of a mutation in ABCC8 or KCNJ11. Am J Pathol (2001) 158:2177–84. doi: 10.1016/S0002-9440(10)64689-5 PMC1891997 11395395

[23] Mohnike K Wieland I Barthlen W Vogelgesang S Empting S Mohnike W . Clinical and genetic evaluation of patients with K_ATP_ channel mutations from the German registry for congenital hyperinsulinism. Horm Res Paediatr (2014) 81:156–68. doi: 10.1159/000356905 24401662

[24] Zawada AM Rogacev KS Müller S Rotter B Winter P Fliser D . Massive analysis of cDNA ends (MACE) and miRNA expression profiling identifies proatherogenic pathways in chronic kidney disease. Epigenetics (2014) 9:161–72. doi: 10.4161/epi.26931 PMC3928179 24184689

[25] Popp MW Maquat LE . Organizing principles of mammalian nonsense-mediated mRNA decay. Annu Rev Genet (2013) 47:139–65. doi: 10.1146/annurev-genet-111212-133424 PMC4148824 24274751

[26] Lee BH Lee J Kim J-M Kang M Kim G-H Choi J-H . Three novel pathogenic mutations in KATP channel genes and somatic imprinting alterations of the 11p15 region in pancreatic tissue in patients with congenital hyperinsulinism. Horm Res Paediatr (2015) 83:204–10. doi: 10.1159/000371445 25765446

[27] Makishima H Maciejewski JP . Pathogenesis and consequences of uniparental disomy in cancer. Clin Cancer Res (2011) 17:3913–23. doi: 10.1158/1078-0432.CCR-10-2900 PMC3523887 21518781

[28] Glaser B Blech I Krakinovsky Y Ekstein J Gillis D Mazor-Aronovitch K . ABCC8 mutation allele frequency in the ashkenazi Jewish population and risk of focal hyperinsulinemic hypoglycemia. Genet Med (2011) 13:891–4. doi: 10.1097/GIM.0b013e31821fea33 21716120

[29] Badens C Mattei MG Imbert AM Lapouméroulie C Martini N Michel G . A novel mechansims for thalassemia intermedia. Lancet (2002) 359:132–3. doi: 10.1016/s0140-6736(02)07338-5 11809258

[30] Vinatier I Martin X Costa JM Bazin A Giraudier S Joly P . A late onset sickle cell disease reveals a mosaic segmental uniparental isodisomy of chromosome 11p15. Blood Cells Mol Dis (2015) 54:53–5. doi: 10.1016/j.bcmd.2014.07.021 25159120

[31] Jubb AM Chalasani S Frantz GD Smits R Grasch HI Kavi V . Achaete-scute like 2 (ascl2) is a target of wnt signaling and is upregulated in intestinal neoplasia. Oncogene (2006) 25:3445–57. doi: 10.1038/sj.onc.1209382 16568095

[32] Tunster SJ Van de Pette M Creeth HDJ Lefebvre L John RM . Fetal growth restriction in a genetic model of sporadic beckwith-wiedemann syndrome. Dis Model Mech (2018) 11:pii: dmm035832. doi: 10.1242/dmm.035832 PMC6262809 30158284

[33] Jennings RE, Berry AA, Strutt JP, Gerrard DT, Hanley NA. human pancreas development. Development (2015) 142:3126–37. doi: 10.1242/dev.120063 26395141

